# CT-based three-dimensional morphometric analysis of the bony palate and hard palatal mucosa in female orthognathic surgery candidates

**DOI:** 10.3389/froh.2026.1763657

**Published:** 2026-05-12

**Authors:** Yusuke Suzuki, Shintaro Kondo, Sho Kuroe, Shinichi Negishi

**Affiliations:** 1 School of Dentistry at Matsudo, Department of Orthodontics, Nihon University, Chiba, Japan; 2 Research Institute of Oral Science, School of Dentistry at Matsudo, Nihon University, Chiba, Japan

**Keywords:** anatomy, computed tomography, Japanese, liner-measurement, orthodontic treatment, palatal mucosa, palate

## Abstract

**Introduction:**

Morphological studies of the bony palate remain limited. This study aimed to investigate the three-dimensional relationship between the bony palate and the hard palatal mucosa, as well as palatal dimensional differences according to anteroposterior jaw relationships, using CT data.

**Methods:**

CT data from 116 female patients (95 young adults; 15–29 years, 21 middle-aged adults; 30–56 years) who required orthodontic treatment combined with orthognathic surgery were analyzed morphometrically using medical image-processing software. The anteroposterior relationship between the maxilla and mandible was determined using lateral cephalograms, and participants were divided into three groups: Class I (normal maxillomandibular relationship), Class II (maxilla positioned anterior to the mandible), and Class III (mandible positioned anterior to the maxilla). Intergroup palatal dimensional differences were analyzed using non-parametric tests (Wilcoxon test and Steel–Dwass test).

**Results:**

Palatal mucosal thickness in the first molar region (PMT) showed substantial inter-individual variation, with no significant age-related differences. Palatal width was narrower, whereas palatal length was greater in Class II than in Class III. Palatal depth was greater in Class II than that in Classes I and III at the first premolar (P1), but no intergroup difference was observed at the first molar (M1).

**Conclusion:**

PMT showed no age-related differences and may serve as a representative indicator of palatal mucosal structure. In Class II, the bony palate was narrower and deeper in the P1 region, whereas the palatal depth in the M1 region did not differ significantly from that in Classes I and III.

## 1 Introduction

The anterior major region of the palate is the hard palate; the submucosa is lined by the palatine process of the maxilla and horizontal plate of the palatine bone, and the mucosa has restricted mobility. The posterior minor region is the soft palate; the palatine muscles are present submucosally, and the mucosa has high mobility (1). The hard palate is covered by masticatory mucosa, whereas the soft palate is covered by lining mucosa; they serve different functions. The hard palate contributes to mastication, bolus formation, and articulation, while the soft palate plays a critical role in nasopharyngeal and oropharyngeal closure during swallowing, as well as speech production. According to the process model of feeding and swallowing, these palatal functions facilitate the smooth transition from stage I transport to processing, stage II transport, and the pharyngeal phase. These phases are indispensable for efficient mastication, bolus control, and safe swallowing (2, 3).

Furthermore, morphological characteristics of the hard palate have been linked to articulation. A higher palatal vault has been shown to alter speech sounds (4). In addition, patients requiring orthodontic treatment combined with orthognathic surgery for maxillary protrusion exhibit a higher frequency of articulation disorders than those with normal anteroposterior jaw relationships (5).

Studies using dental casts have demonstrated that patients with maxillary protrusion exhibit a narrower and deeper palate (6, 7), whereas those with mandibular protrusion present with a wider and shallower palate (8, 9). In orthodontics, the anteroposterior jaw relationship is a fundamental diagnostic parameter guiding treatment decisions. However, despite its clinical importance, three-dimensional morphological studies comparing bony palate across different anteroposterior jaw relationships remain scarce.

The mucosal thickness of the hard palate has primarily been investigated in periodontology to determine suitable graft sites for gingival augmentation procedures. Probe-based measurements were historically common, but these methods require local anesthesia and are susceptible to operator-dependent variation (10). Consequently, CT-based methods—capable of simultaneously visualizing bone and soft tissue with high reproducibility—have become widely adopted (11). More recently, studies have attempted to integrate CT data with intraoral scanner-derived surface data to analyze the three-dimensional relationship between hard and soft tissues (12). Although differences in palatal mucosal thickness by sex, age (12), and location (11) have been reported, no consensus has been reached. It has also been reported that palatal mucosa thickens with age and shows considerable individual variation, and several studies have reported sex-related differences (11, 12).

From an orthodontic perspective, research has often focused on identifying optimal insertion sites for temporary anchorage devices (TADs) based on mucosal thickness and available bone (13). However, most studies rarely account for the difference in bony palate structure, and investigations evaluating the relationship between bony palate shape and mucosal thickness remain limited.

Orthodontic treatment combined with orthognathic surgery is indicated for patients with severe anteroposterior, vertical, or transverse skeletal discrepancies in whom orthodontic treatment alone cannot achieve normal occlusion. The skeletal diagnosis is typically established using lateral cephalometric radiographs (14). Among patients with a normal sagittal relationship (Class I), surgery is required when substantial vertical or transverse discrepancies, such as a gummy smile, open bite, or facial asymmetry, are present. Class II represents maxillary protrusion or mandibular retrusion, whereas Class III corresponds to mandibular protrusion or maxillary retrusion. Because patients requiring orthognathic surgery exhibit greater skeletal deviations than those managed with orthodontic treatment alone, their craniofacial and palatal morphology may show more pronounced structural characteristics.

Reports in periodontal surgery have indicated that the mucosal thickness at donor sites on the alveolar ridge decreases as palatal depth increases (15, 16), suggesting a possible association between palatal morphology and mucosal thickness. However, studies assessing mucosal thickness at the palatal vault floor are scarce. Even in investigations that measured mucosal thickness in the palatal vault region to determine optimal insertion sites for TADs, the morphology of the bony palate was not considered (17).

Regarding the relationship between skeletal anteroposterior jaw relationships and palatal morphology, previous studies have reported a negative correlation between palatal width and palatal depth—wider palates tend to be shallower, whereas narrower palates tend to be deeper (9, 18). A positive correlation between palatal width and palatal length has also been demonstrated (19). These findings imply that characteristic bony palatal structure may exist for each anteroposterior jaw relationship and may potentially influence palatal mucosal thickness. Nonetheless, most previous studies have not specifically analyzed bony palatal morphology nor examined these relationships within the context of Class I–III classifications.

These previous reports have led to the commonly held belief that patients with Class II tend to have a narrow and deep palate, whereas those with Class III tend to exhibit a wide and shallow palate. However, in clinical practice, we frequently encounter cases that do not conform to this belief. Therefore, the present study aimed to investigate this relationship by examining patients undergoing orthognathic surgery, who typically present with more pronounced skeletal discrepancies. In this study, we used medical CT data to perform a three-dimensional analysis of palatal mucosal thickness and bony palate morphology and clarified the relationship between bony palate morphology and palatal mucosal thickness, as well as the relationship between the anteroposterior jaw relationship (Class I to III) and bony palate morphology.

## 2 Materials and methods

### 2.1 Participants and 3D-data

**Figure 1:**
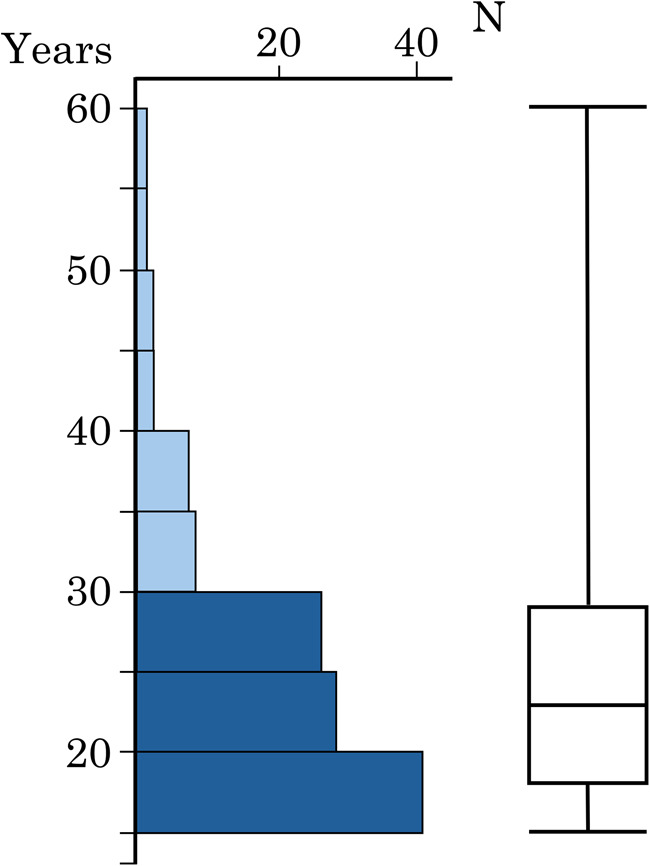
Distribution of participant age (years) with histogram and box plot. The histogram shows the age distribution of the participants. Dark bars indicate young adult group (15—29 years), and light bars indicate middle-aged group (30—56 years). The box plot shows the first and third quartiles (box edges), median (line), and range (whiskers).

The present study included 116 female patients who visited Nihon University School of Dentistry at Matsudo Dental Hospital and were diagnosed as requiring orthognathic surgery in combination with orthodontic treatment because of marked anteroposterior and vertical skeletal discrepancies that made orthodontic treatment alone insufficient. The patients were 15–56 years (mean age, 24.3 ± 8.2 years), and most were under 30 years. Adolescent participants were determined to have completed further rapid skeletal growth based on hand–wrist radiographic assessment. To examine whether age could be a confounding factor, participants were divided two age groups; under 30 years (young adult group; 15–29 years), who accounted for 88.2% of the total participants, and those aged 30 years or older (middle-aged group; 30–56 years) (Figure 1).

Exclusion criteria were cleft lip and/or palate, traumatic facial injury, syndromic craniofacial deformity, systemic conditions known to influence soft tissue characteristics, clinically significant periodontal disease, a history of orthognathic or other corrective jaw surgery, and congenital absence of two or more teeth excluding the third molars. Patients with severe facial asymmetry were also excluded. Severe asymmetry was defined as a deviation of the *menton* (Me) greater than 4 mm from the facial midline on frontal cephalometric radiographs; the facial midline was defined as the line connecting the *crista galli* (Cg) and the *anterior nasal spine* (ANS). In addition, patients in whom the tongue contacted the palatal mucosa at the measurement site during CT acquisition and in whom the boundary between the palatal mucosa and the tongue could not be clearly identified were excluded (20).

CT image data (DICOM format) obtained at the initial visit, before orthognathic surgery, were used for analysis. CT imaging was performed with a 64-slice multi-detector CT system (Aquilion 64, Toshiba Medical Systems, Japan). The imaging conditions were a tube voltage of 120 kV, tube current of 100 mA, and slice thickness of 0.5 mm. CT images were acquired in the axial plane and oriented with reference to the occlusal plane. This imaging method is routinely employed to minimize artefacts caused by dental crowns. CT imaging was conducted as part of the standard clinical workflow for orthognathic surgical planning at our institution. Imaging parameters were selected in accordance with institutional protocols to ensure diagnostic adequacy while minimizing radiation exposure. No additional imaging was performed for research purposes. CBCT or MDCT imaging is routinely performed as a preoperative screening modality; although CBCT involves a lower radiation dose, MDCT was chosen when comprehensive evaluation including soft tissue was required. Although MDCT involves a higher radiation dose than CBCT, imaging was performed within clinically acceptable limits. In this study, MDCT data, which can visualize soft tissue, were selected to measure mucosal thickness.

### 2.2 Anteroposterior relationship between the maxilla and mandible

**Figure 2:**
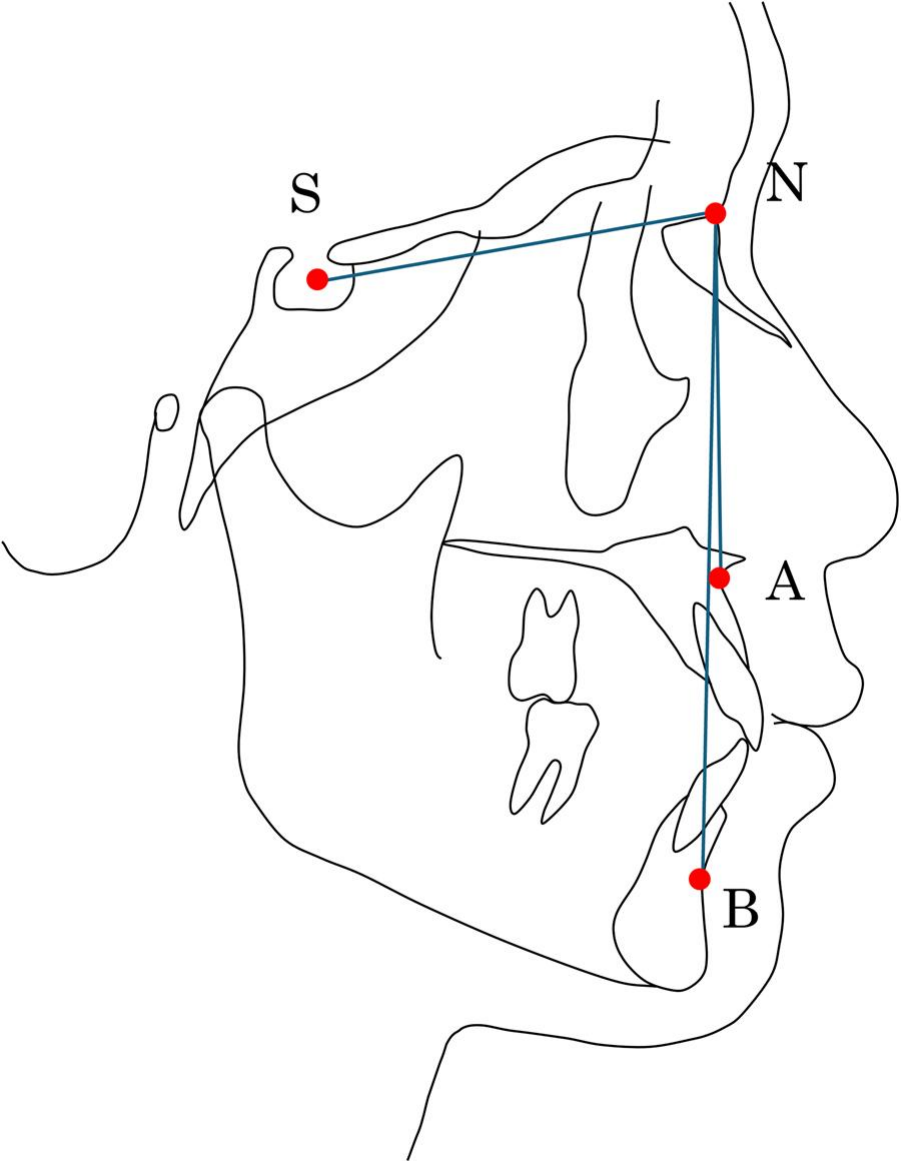
ANB angle on the lateral cephalogram. The ANB angle represents the anteroposterior jaw relationship. In practice, ANB angle is calculated by subtracting SNB angle from SNA angle. Skeletal classifications are defined as follows: Class I (1.5° ≤ ANB angle ≤ 4.0°), Class II (ANB angle > 4.0°), and Class III (ANB angle < 1.5°). S, *sella turcica*; N, *nasion*; A, point A; B, point B.

Skeletal anteroposterior jaw relationships were diagnosed on lateral cephalometric radiographs. In accordance with Seo, H. J. et al. (14), the ANB angle (calculated as SNA – SNB; A; point A, B; point B, N; *nasion*, S; *sella turcica*) was used to classify the sagittal maxillomandibular relationship (Figure 2). A normal anteroposterior jaw relationship between the maxilla and mandible was defined as Class I (1.5° ≤ ANB ≤ 4.0°). Cases in which the maxilla was positioned significantly anterior to the mandible were classified as Class II (ANB > 4.0°), and cases in which the mandible was positioned significantly anterior to the maxilla were classified as Class III (ANB < 1.5°) (Table 1). In this study, the vertical skeletal pattern was not analyzed because the sample size within each anteroposterior relationship group was insufficient.

**Table 1:** Number of samples in each skeletal relation based on ANB angle.

Class I 1.5° ≤ANB≤4.0°		Class2 (ANB > 4.0°)		Class3 (ANB < 1.5°)		Total
%	(*n*)	%	(*n*)	%	(*n*)	*N*
17.2%	(20)	31.9%	(37)	50.9%	(59)	116

This study was approved by the Ethics Review Committee of Nihon University School of Dentistry at Matsudo (Approval No. EC 23-020).

### 2.3 Measurement methods

#### 2.3.1 Measurement software and methods

Linear distances and areas were measured using medical image-processing software (Mimics 27.0, Materialise, Belgium). To distinguish soft tissue from hard tissue, the gray-scale threshold was manually adjusted, and palatal mucosal thickness and bony palatal morphology were measured on the resulting images.

#### 2.3.2 Measurement parameters

The following parameters were measured: palatal mucosal thickness, and the width, depth, length, and frontal cross-sectional area of the bony palate. Palatal width, palatal depth, and frontal (coronal) cross-sectional area were evaluated at the anterior palate (first premolar region, P1) and at the first molar region (M1), where palatal width and depth reach their maximum within the palate.

**Figure 3:**
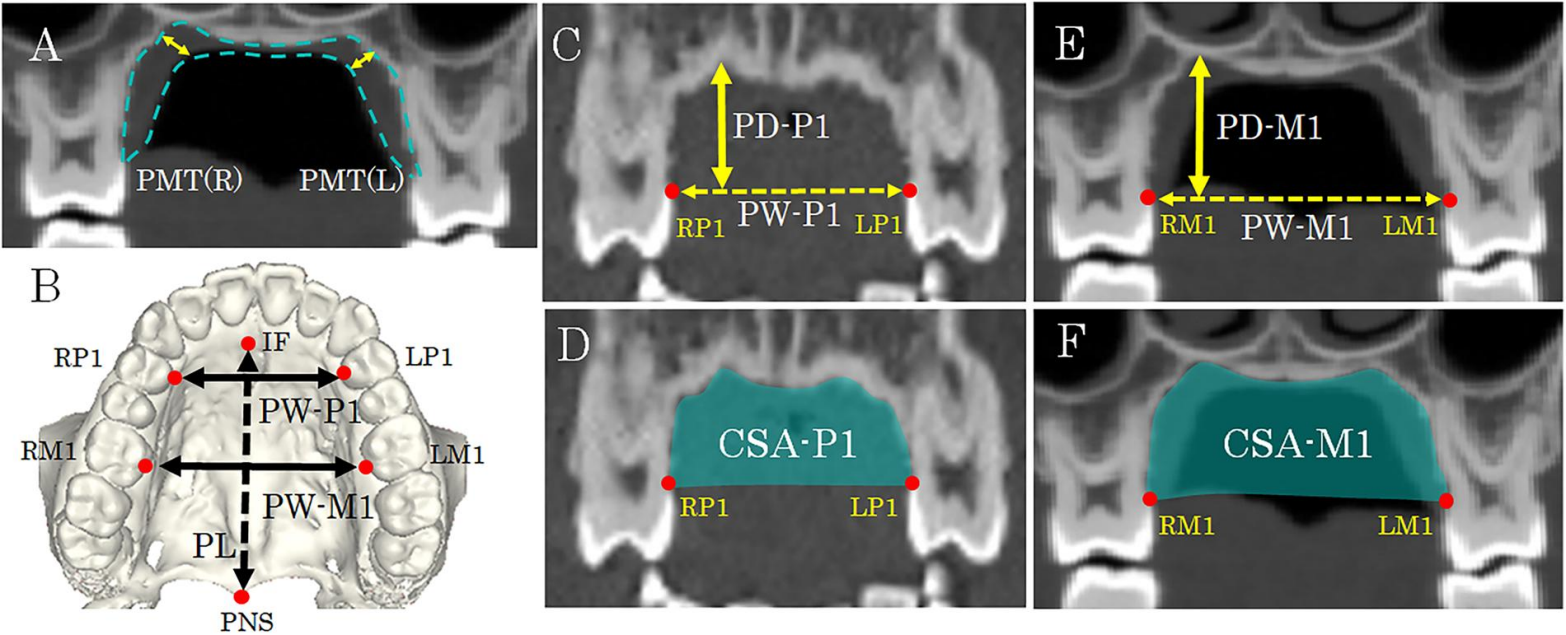
Measurement sites for palatal mucosal thickness and bony palate morphology. **(A)** Frontal section at the M1 region. Yellow lines indicate measurement sites for palatal mucosal thickness (PMT), recorded bilaterally [PMT(R) and PMT(L)]. Dashed line shows palatal mucosa area. **(B)** Occlusal view of the 3D volume-rendered model. Solid lines show measurement sites for palatal widths at P1 and M1 (PW-P1 and PW-M1). The dashed line indicates palatal length (PL). **(C)** Frontal section at P1 showing palatal width (PW-P1; dashed line) and palatal depth (PD-P1; solid line). **(D)** Frontal section at P1 showing the frontal cross-sectional area (CSA-P1; green region). **(E)** Frontal section at M1 showing palatal width (PW-M1; dashed line) and palatal depth (PD-M1; solid line). **(F)** Frontal section at M1 showing the frontal cross-sectional area (CSA-M1; green region). Red circles indicate measurement landmarks: IF, anterior edge of the incisive fossa; RP1/LP1, right/left maxillary first premolar; RM1/LM1, right/left maxillary first molar (cervical enamel–bone junction); PNS; posterior nasal spine.

Palatal mucosal thickness (PMT): On frontal sections passing through the mesiodistal midpoint of M1, palatal mucosal thickness was defined as the perpendicular distance from the point—where the horizontal plane of the bony palate meets the rising surface of the alveolar process—to the surface of the palatal mucosa (Figure 3A). Measurements were taken bilaterally.

Palatal width at P1 and M1 (PW-P1, PW-M1): Palatal width was measured on frontal sections as the distance between the junctions of the lingual enamel surface and the maxillary bone at the mesiodistal midpoint of the crowns of the bilateral P1s (PW-P1) and M1s (PW-M1) (Figures 3B, C, E).

Palatal depth at the first premolar and first molar (PD-P1, PD-M1): Palatal depth was measured as the distance from the line connecting the enamel–bone junction points of the bilateral P1s or M1s to the deepest point of the palatal vault, along a line drawn perpendicular to this reference line, on frontal sections (Figures 3C–E).

Frontal cross-sectional area at P1 and M1 (CSA-P1, CSA-M1): The frontal cross-sectional area at P1 and M1 was measured on frontal sections that included the line connecting the enamel–bone junction points at the mesiodistal midpoints of the bilateral P1 (CSA-P1) and M1 (CSA-M1) (Figures 3D–F).

Palatal length (PL): Palatal length was defined as the distance between the anterior margin of the incisive fossa and the PNS (*posterior nasal spine*), measured on volume-rendered three-dimensional images (Figure 3B).

Based on these measurements, the following areas, volumes, and indices were calculated:

Palatalarea(PA)=PW−M1×PL

Palatalvolume(PV)=PW−M1×PL×PD−M1

Width–lengthindex(WLindex)=(PW−M1/PL)×100

Depth−widthindex(DWindex)=(PD−M1/PW−M1)×100

PWindex=(PW−P1/PW−M1)×100

PDindex=(PD−P1/PD−M1)×100

CSAindex=(CSA−P1/CSA−M1)×100

#### 2.3.3 Statistical analysis

In this study, the variation in each measurement was large, so nonparametric procedures were used for the analysis. Left–right differences in palatal mucosal thickness were assessed using the Wilcoxon signed-rank test. The Wilcoxon test was used to compare the two age groups. Comparisons among the three classes were performed using the Steel–Dwass test, which controls for multiple comparisons. Spearman's rank correlation coefficients were calculated. Statistical analyses were conducted using SPSS software (IBM SPSS Statistics for Windows, version 31.0; IBM Corp., Armonk, NY, USA).

#### 2.3.4 Reliability of measurements

To assess intra-observer reliability, 50 CT datasets were randomly selected, and all measurements were re-measured by the same examiner (YS) after an interval of >2 months. The random error of a single determination was estimated using Dahlberg's formula where d is the difference between the first and second measurements, and N is the number of double-measured cases (single determinations) (21).

$$\mathrm{error}=\sqrt{\frac{\sum d^{2}}{2N}}$$

## 3 Results

### 3.1 Reliability of measurements

For both linear and area measurements, no significant differences were found between the first and second measurements using the paired *t*-test. Therefore, no systematic methodological error was detected. The differences between the mean values of the first and second measurements ranged from 0.3–0.1 mm for distance, and 0.0–5.8 mm^2^ for area. The intra-observer error calculated using Dahlberg's formula was 0.0–0.3 mm. The error variance accounted for 0.4–0.6% of the total variance for distance measurements and 4.2–5.8% for area measurements. These findings indicate that random error was sufficiently small compared with the total variance of the measurements, and it is unlikely that measurement error biased the results of the statistical analyses.

### 3.2 Palatal mucosal thickness

Descriptive statistics for palatal mucosal thickness (PMT) on the right and left sides are shown in Table 2. PMT ranged from 1.8 to 6.4 mm, indicating a wide range and substantial inter-individual variation. As no significant left–right difference was detected, the mean of both sides was used as the representative value for each participant in subsequent analyses (Table 2). No age-related differences were observed in either the median or range (Table 3).

**Table 2:** Palatal mucosal thickness on the right and left sides in mm (*N* = 116).

Palatal side	Median	(IQR)	(Min - Max)	
Right side	3.7	(3.1–4.4)	(1.9–6.4)	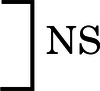
Left side	3.6	(3.1–4.2)	(1.8–5.9)	

IQR, interquartile range. Difference between the left and right sides was not significant by the signed Wilcoxon test.

**Table 3:** Palatal mucosal thickness on the two age groups in mm. .

Age group	*n*	Median	(IQR)	(Min - Max)	
15 years old or older but under 30 years old	95	3.7	(3.1–4.2)	(2.0–5.6)	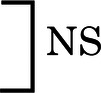
30 years or older	21	3.7	(3.2–4.1)	(2.1–5.6)	

IQR, interquartile range. Difference between the two age groups was not significant by the Wilcoxon test.

### 3.3 Comparison of hard palate size and mucosal thickness according to maxillomandibular relationship

**Figure 4:**
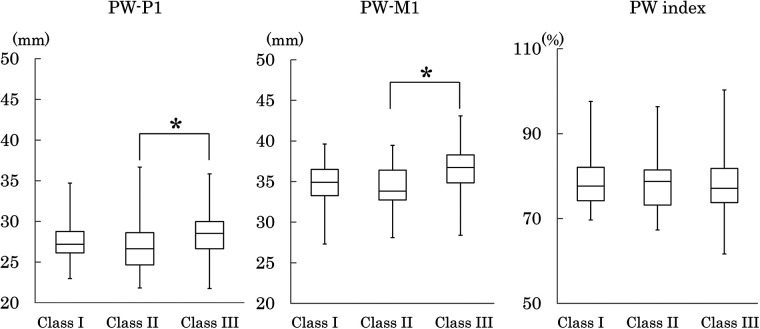
Palatal width at the P1 (PW-P1) and M1 (PW-M1) regions, and its ratio (PW index). Box-and-whisker plots show the first and third quartiles (box edges), the median (line), and the range (whiskers). Inter-class differences were assessed using the Steel–Dwass test. *p < 0.05.

**Figure 5:**
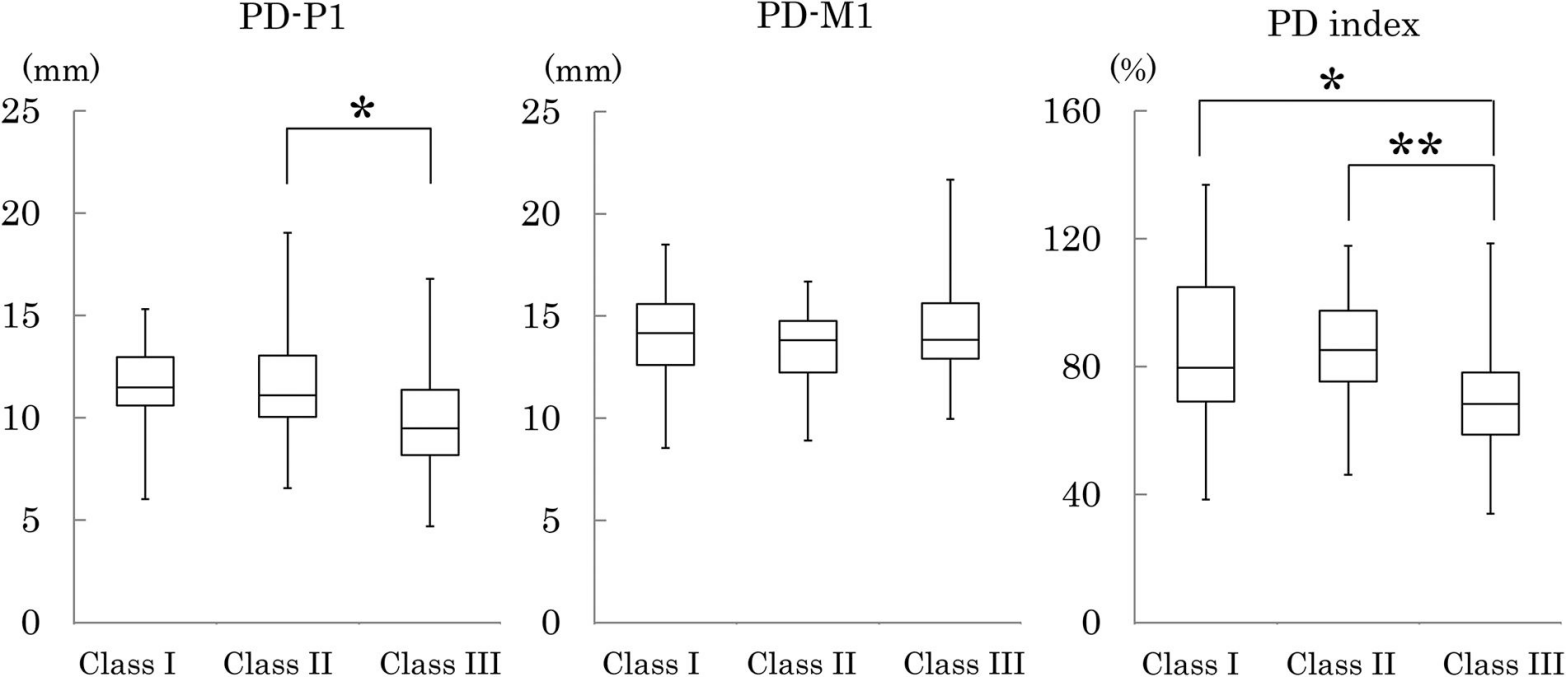
Palatal depth at the P1 (PD-P1) and M1 (PD-M1) regions, and its ratio (PD index). Plot conventions are identical to Figure 3. Inter-class differences were assessed using the Steel–Dwass test. *p < 0.05; **p < 0.01.

**Figure 6:**
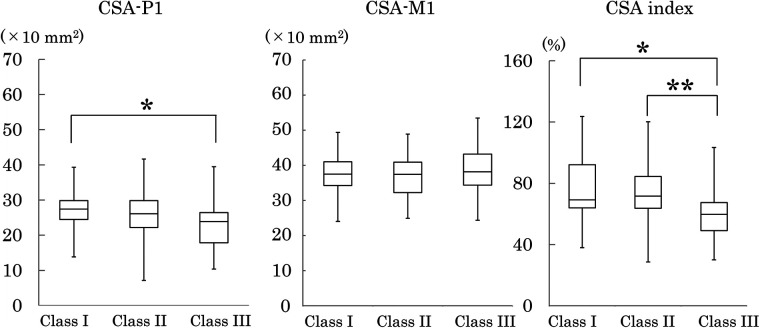
Frontal cross-sectional area at the P1 (CSA-P1) and M1 (CSA-M1) regions, and its ratio (CSA index). Plot conventions are identical to Figure 3. Inter-class differences were assessed using the Steel–Dwass test. *p < 0.05; **p < 0.01.

**Figure 7:**
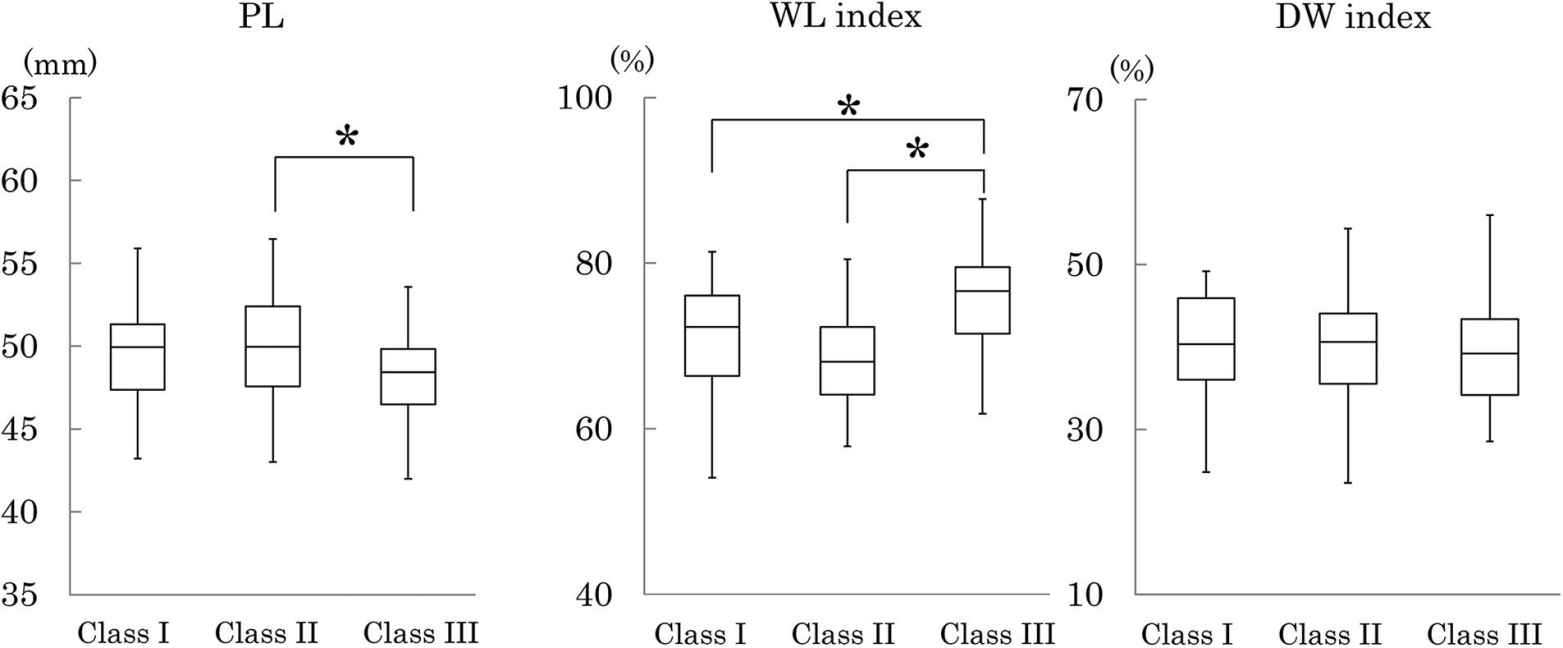
Palatal length (PL) and width–length index (PW-M1/PL). Plot conventions are identical to Figure 3. Inter-class differences were assessed using the Steel–Dwass test. *p < 0.05.

**Figure 8:**
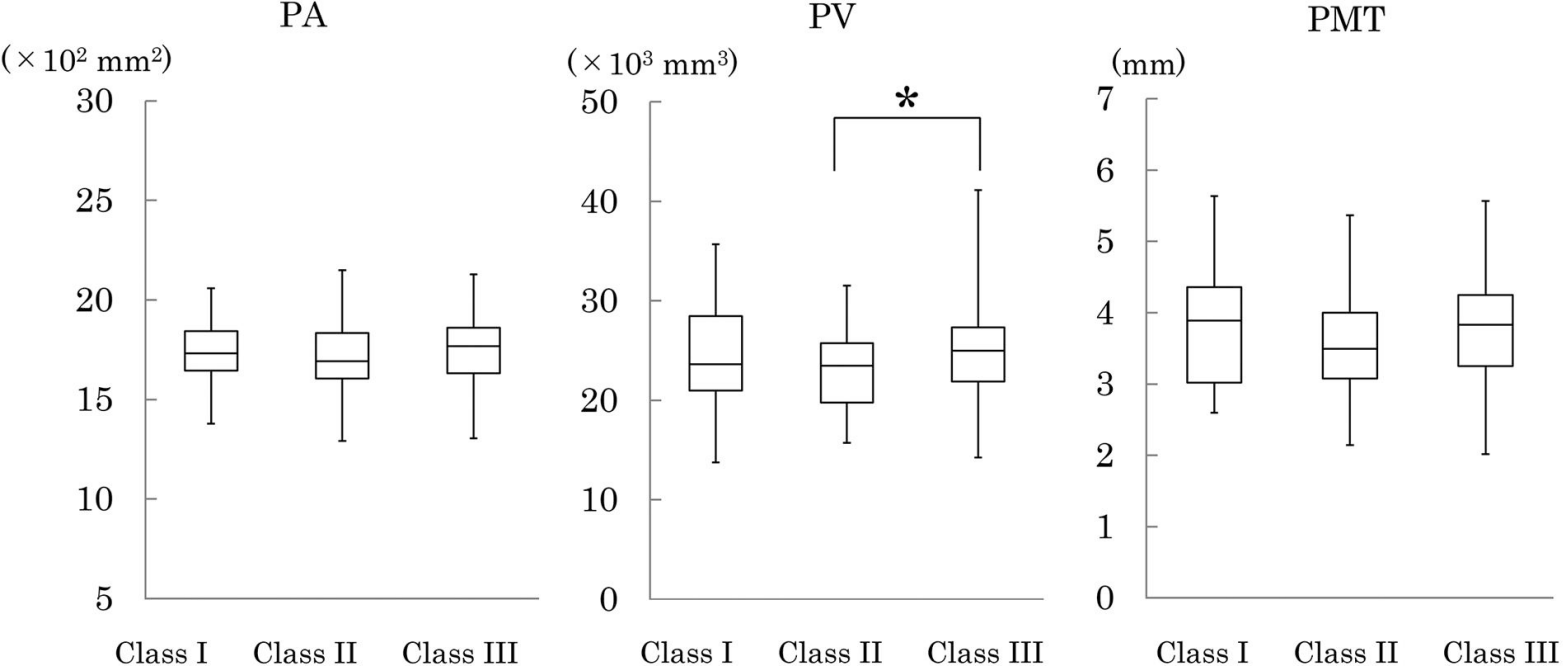
Palatal area (PA), palatal volume (PV), and palatal mucosal thickness (PMT). Plot conventions are identical to Figure 3. Inter-class differences were assessed using the Steel–Dwass test. *p < 0.05.

 Table 4 shows the measured values and indices of the bony palate, together with the descriptive statistics for palatal mucosal thickness. Bony palate dimensions and palatal mucosal thickness were compared among the three skeletal classes based on the maxillomandibular relationship (Figures 4–8).

**Table 4:** Basic statistics of the palatal structures and indices (*N* = 116). .

Measurement/index items	Abbreviation	Unit	Median	IQR	Min	Max	Range
Palatal width at P1	PW-P1	(mm)	27.5	25.9– 29.5	21.7	36.7	14.9
Palatal width at M1	PW-M1	(mm)	35.4	33.6– 37.9	27.3	43.1	15.8
Maximum palatal depth at P1	PD-P1	(mm)	10.8	8.8– 12.0	4.7	19.0	14.3
Maximum palatal depth at M1	PD-M1	(mm)	13.8	12.7–14.9	8.5	21.7	13.1
Cross-sectional area at P1	CSA-P1	(×10 mm^2^)	24.8	19.9–28.6	7.1	41.6	34.5
Cross-sectional area at M1	CSA-M1	(×10 mm^2^)	37.6	33.2–42.0	24.0	53.5	29.5
Palatal length (from the anterior end of incisal fossa to PNS)	PL	(mm)	49.1	47.0–50.8	42.0	56.5	14.5
Paratal area (PL × PW-M1)	PA	(×10^2^ mm^2^)	17.4	16.1–18.5	12.9	21.5	8.6
Palatal volume (PW-M1 × PL × PD-M1)	PV	(×10^3^ mm^3^)	24.1	21.3–27.1	13.8	41.1	27.4
Width-length index (PW-M1/PL ×100)	WL index	(%)	72.5	67.5–78.1	54.1	87.8	33.7
Depth-width index (PD-M1/PW-M1 × 100)	DW index	(%)	39.7	35.3–44.2	23.5	56.0	32.5
PW-P1/PW-M1 × 100	PW index	(%)	77.5	73.6–81.7	61.6	100.3	38.7
PD-P1/PD- M1 × 100	PD index	(%)	75.9	63.2–87.2	34.0	136.9	102.9
CSA-P1/CSA-M1 × 100	CSA index	(%)	65.2	53.5–74.5	28.6	123.6	94.9
Palatal mucosa thickness (average of left and right sides)	PMT	(mm)	3.7	3.1–4.2	2.0	5.6	3.6

P1, first premolar; M1, first molar; PNS, posterior nasal spain; IQR, interquartile range.

#### 3.3.1 Palatal width

Palatal width (PW) at both the P1 and M1 regions was significantly greater in Class III than in Class II. The index expressing palatal width at M1 relative to that at P1 (PW index) showed no significant differences among the three classes (Figure 4).

#### 3.3.2 Palatal depth

Palatal depth (PD) at P1 was significantly shallower in Class III than in Class II, but no significant differences among the three classes were observed at M1. The depth index (PD index) was significantly smaller in Class III than in Classes I and II, indicating that palatal depth at the P1 region was relatively shallow in Class III (Figure 5).

#### 3.3.3 Frontal cross-sectional area

The frontal cross-sectional area (CSA) at P1 was significantly smaller in Class III than Class I. No significant differences among the three classes were observed in the cross-sectional area at M1. The index of cross-sectional area at P1 relative to that at M1 was significantly smaller in Class III than in Classes I and II, indicating a smaller cross-sectional area in the P1 region in Class III (Figure 6).

#### 3.3.4 Palatal length

Palatal length (PL) was significantly shorter in Class III than Class II (Figure 7).

#### 3.3.5 Width–length index (WL index)

The Width-length index (WL index) was significantly larger in Class III than in Classes I and II. This finding indicates that palatal width was relatively greater in Class III than in the other classes (Figure 7).

#### 3.3.6 Depth-width index (DW index)

The Depth-width index (DW index) showed no significant differences among the three Classes. This result showed that there was no significant inter-class difference in the relative palatal depth at the deepest point relative to the palatal width (Figure 7).

#### 3.3.7 Palatal area and palatal volume

Because palatal width and depth at M1 represent the maximum dimensions of the palate, these values were used to calculate palatal area (PA) and volume (PV). PA did not differ significantly among the three classes. PV, however, was significantly greater in Class III than in Class II. Class III also showed a broader distribution with high-end outliers, indicating greater variability in overall palatal size (Figure 8).

#### 3.3.8 Palatal mucosal thickness

No significant differences in palatal mucosal thickness (PMT) were observed among the three classes. All classes exhibited wide ranges, demonstrating marked individual variability (Figure 8).

### 3.4 Correlation coefficients

Correlation coefficients among the linear measurements are shown in Table 5. Overall, correlations between variables were generally low, however, several significant relationships were observed. Palatal widths at P1 and M1 were positively correlated with each other, as were palatal depths at P1 and M1. By contrast, palatal width at M1 showed a negative correlation with palatal depth at P1. PMT exhibited a weak positive correlation with PD-P1.

**Table 5:** Correlation coefficients between palatal measurements (*N* = 116).

Spearman's rank correlation matrix						
Variables	PW-P1	PW-M1	PD-P1	PD-M1	PL	PMT
PW-P1		0.510	−0.115	−0.067	0.087	−0.038
PW-M1			−0.287	0.042	0.031	−0.109
PD-P1				0.353	0.120	0.218
PD-M1					0.182	0.120
PL						−0.115
PMT						
Exact *p*-values for the correlation matrix						
Variables	PW-P1	PW-M1	PD-P1	PD-M1	PL	PMT
PW-P1		<.0001	0.218	0.478	0.354	0.689
PW-M1			0.002	0.654	0.743	0.246
PD-P1				0.000	0.199	0.019
PD-M1					0.050	0.198
PL						0.217
PMT						

Red indicates *p* < 0.01, and blue indicates *p* < 0.05.

### 3.5 Summary of the results

**Figure 9:**
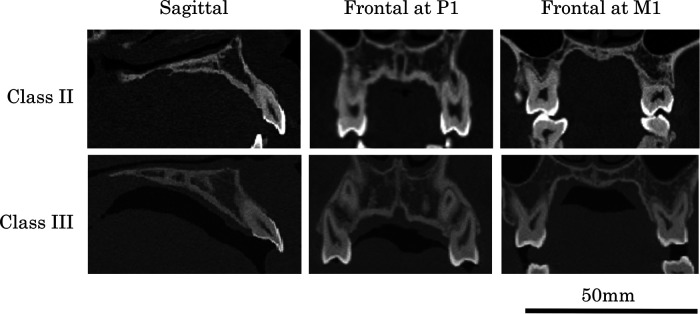
Sagittal and frontal section images at P1 and M1 in typical classes II and III individuals.

**Figure 10:**
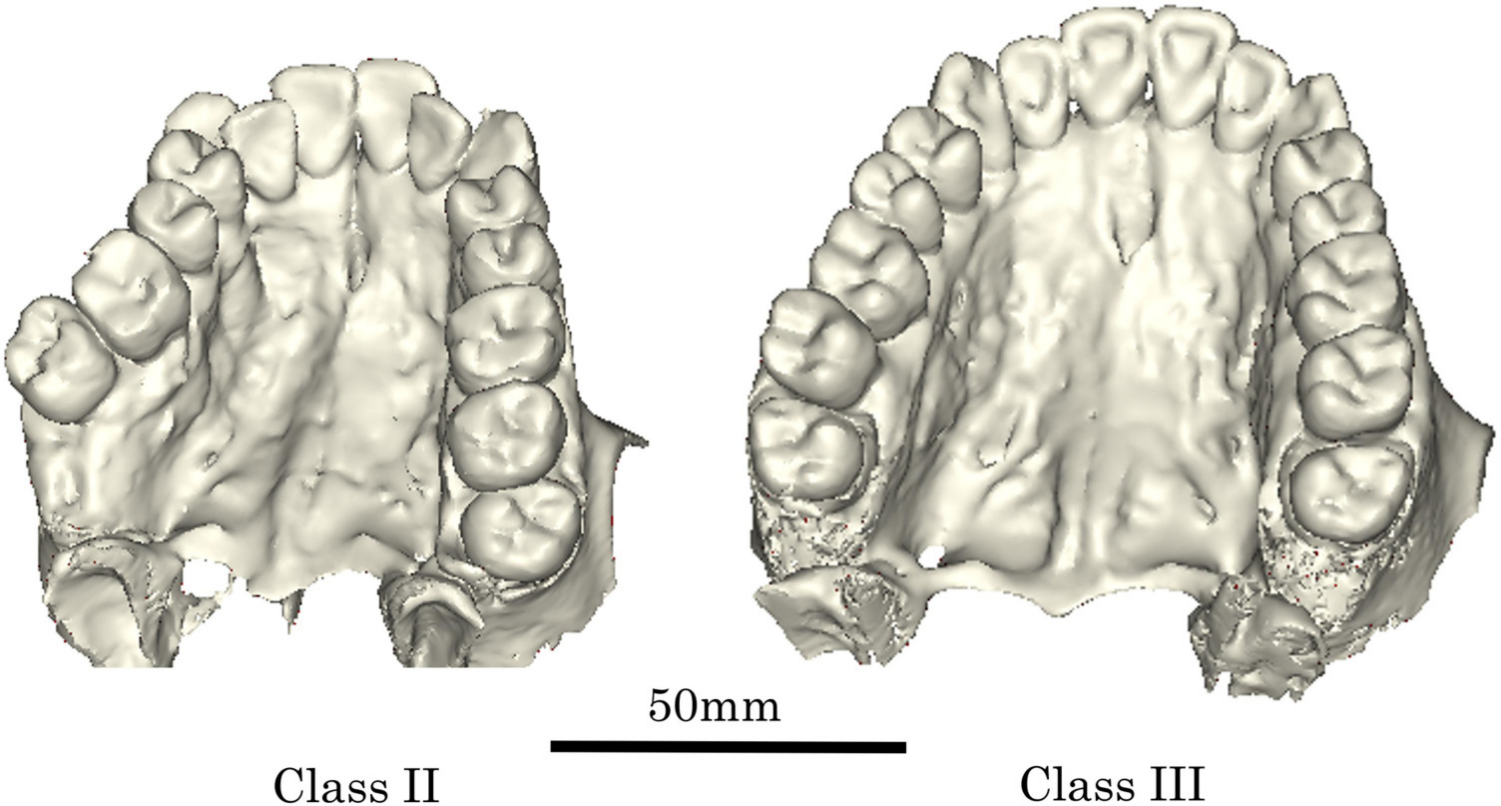
Volume-rendered images in typical classes II and III individuals (the same individuals shown in Figure 8). Volume-rendered images have been drawn with medical image-processing software (Mimics 27.0, Materialise, Belgium).

Differences in bony palatal size and shape between Classes II and III are summarized as follows. Representative sagittal and frontal (P1 and M1) section images, together with volume-rendered models, are shown in Figures 9, 10. Class II displayed a narrower and longer palate compared with Class III. PD-P1 was greater in Class II than in Class III, whereas no inter-class difference was identified PD-M1. In sagittal sections, Class III palates exhibited a gradual increase in depth from anterior to posterior, whereas Class II demonstrated a steep drop at the premolar region followed by a relatively horizontal posterior region. Overall, Class II demonstrated the smallest palatal dimensions among the three groups, despite having greater length, due to the combination of narrow width and a deeper anterior vault.

## 4 Discussion

### 4.1 Participants

This study included patients diagnosed as requiring orthognathic surgery in combination with orthodontic treatment, for whom orthodontic treatment alone was deemed insufficient. The proportion of participants was highest for Class III, followed by Classes II and I (Table 1). The high prevalence of Class III in the Japanese population of this study is consistent with findings from other Asian populations (e.g., Korean) (22) and Caucasian populations (23). In recent years, advances in treatment techniques have enabled many cases of Class II that previously required orthognathic surgery to be managed without surgery (23). Although the frequency of Class II cases was relatively high in this study compared with some previous reports, the proportion of Class II patients requiring orthognathic surgery may decrease in the future. Consequently, changes in the class distribution of patients undergoing orthognathic surgery may also be observed in Japanese population.

### 4.2 Palatal mucosal thickness

Most studies measuring hard palatal mucosal thickness have focused on the maxillary alveolar mucosa to determine autogenous graft donor sites in periodontal surgery and have generally reported thinner mucosa than in the present study. Some reports indicate that the second premolar region is the thickest, that males have greater mucosal thickness than females, and that there is no correlation with age (15). Other studies have suggested that regional differences are more pronounced than sex differences, or that age-related differences lead to thicker mucosa in individuals aged ≥35 years (11). Thus, no consistent findings have emerged.

The adolescent patients were diagnosed as having no further rapid skeletal growth based on hand–wrist radiographic assessment. In this study, individuals aged 15 to 29 years were collectively classified as the young adult group. The young adult group included 82.8% of all participants (Figure 1). The remaining participants (30–56 years) were referred to as the middle-aged group. This study revealed no difference in PMT between the two age groups. Periodontal surgery research has shown that the mucosal thickness near the cervical region of the teeth changes with age, and that it may become thinner in people over the age of 35 (11).

Because the participants of this study were female patients who were candidates for orthognathic surgery, it was not possible to study young people still growing or elderly people, so age-related changes throughout the life cycle in the mucosal thickness cannot be determined. Furthermore, it is unclear how vertical skeletal patterns and sexual differences affect mucosal thickness. From these perspectives, the results of this study are limited.

In the present study, CT imaging was performed with the tongue in the resting position. Cases in which the tongue contacted the palate, thereby obscuring the boundary between the palatal mucosa and the tongue, were excluded because accurate measurement of palatal mucosal thickness was not possible. The influence of resting tongue posture has been discussed in previous studies, and in investigations such as the present one—where mucosal thickness is measured solely from CT data—it represents one of the methodological concerns.

The measurement site was selected as the presumed thickest region of the palatal mucosa, considering that the absence of tongue contact and the slice thickness of the CT. As a result, the values obtained in this study were larger than those reported in many previous studies.

Palatal mucosal thickness (PMT) in this study was measured by selecting a region that could be measured stably on CT images. In other words, it was the region least affected by the tongue during imaging. Future research into mapping the palatal mucosa will be necessary, the PMT showed no changes with age, and it may serve as a representative indicator of palatal mucosal structure.

Studies that have examined the placement of orthodontic anchor screws have measured sites comparable to those in this study (13, 17). When observed from a frontal cross section, the thickness of the palatal mucosa in the area corresponding to M1 was approximately 3.9 mm ± 1.1 mm (17). In their study, palatal mucosal thickness was measured in 3-mm intervals, and therefore the maximum thickness was not necessarily captured, nevertheless, their values were relatively close to those obtained in the present study (20). From the perspective of removable denture design, the palatal mucosal thickness in the palatal seal area was measured and reported values ranging from 1.7 to 6.6 mm (24). Their measurement value was almost identical to the range in the present study.

In the present study, no left–right difference in palatal mucosal thickness was observed, although individual variation was clearly substantial. Correlation analysis between palatal size and mucosal thickness revealed no significant correlation for most linear measurements, although a weak positive correlation was found between palatal depth at P1 and mucosal thickness. Therefore, individuals with a deeper palate in the premolar region tended to have thicker mucosa. Analysis according to the anteroposterior jaw relationship showed that Class II had deeper palate in the premolar region, however, no inter-class differences in mucosal thickness were observed.

In summary, the mucosal thickness of the deepest part of the palate (PMT) was less affected by age-related differences, but there was considerable individual variation. A weak positive correlation was observed with palatal depth at P1, but the correlation with palatal size at other parts was weak. Comparison with previous studies suggests that mucosal thickness varies greatly depending on the measurement site (17). Therefore, future research should develop methods to measure site-specific mucosal thickness in more detail.

### 4.3 Bony palatal size and shape

Morphological studies analyzing hard palates using dental casts have reported that narrow palates are associated with a tendency toward a deeper palate (25), suggesting a link between morphological characteristics and functional adaptation. Class II patients frequently exhibit a narrower and deeper palate compared with Class I and Class III patients (26, 27).

Maxillary protrusion (Class II) results in occlusion between the posterior maxilla and anterior mandible, whereas mandibular protrusion (Class III) results in occlusion between the anterior maxilla and posterior mandible. Since the mandible has a U-shaped basal region, the alveolar region is strongly influenced by the shape of the mandibular base. In the maxilla, the bottom surface of the maxillary body is flat, so the shape of the alveolar process is less influenced by the body shape. The mandibular dentition is thought to be more restricted by the basal region of the jawbone than the maxillary dentition. Assuming that the mandibular arch width is constant, maxillary molar arch width tends to be narrower in Class II, whereas maxillary anterior arch width tends to be wider in Class III (28).

Based on this information, it is commonly believed that Class II has a narrow and deep palate, while Class III has a wide and shallow palate. To verify this common belief, we summarized the morphological characteristics of bony palate in Classes II and III (Figures 9, 10).

The overall palatal size (PV) was larger in Class III than in Class II. Class II exhibited a narrower palate at both the P1 and M1 regions than Class III, but the palatal length was longer in Class II. The PD-P1 was greater in Class II than in Class III, but no inter-class difference was observed PD-M1. The results showed that the palatal curvature of Class III was gentle in the sagittal section, whereas the palatal depth of Class II decreased sharply up to the premolar region and then remained nearly horizontal in the molar region (Figures 9, 10).

Class II patients are characterized by a deep anterior palate, rather than a deep palate overall, i.e., the common belief is not necessarily correct. Many studies have reported that Class III patients tend to have wide and shallow palates (9, 18). Furthermore, a negative correlation has been reported between palatal width at M1 and palatal depth at M1, and a positive correlation between palatal width at M1 and palatal length, defined as the distance from the incisive papilla to the PNS (9, 19).

In the present study, a negative correlation was observed between palatal width at M1 and palatal depth at P1 (Table 4), but no correlation was found between palatal width at M1 and palatal depth at M1. Palatal depth at P1 was greater in Class II than in Class III, whereas palatal depth at M1 showed no inter-class differences (Figure 5). Contrary to previous reports, the entire palate could not be described as deeper in Class II. It has been reported that the posterior palatal region is strongly influenced by genetic factors, whereas the anterior region is more susceptible to environmental influences (29). This fact likely explains why inter-class differences appeared predominantly in the anterior region (P1 area).

At present, it remains unclear how the morphometric findings of this study—namely, the differences between anteroposterior relation of jaws and palatal mucosa thickness (PMT), and the three-dimensional structures of the bony palate—may directly contribute to clinical decision-making. In addition, the sample size of this study was not sufficient to allow an analysis of vertical jaw relationships. Further studies incorporating vertical skeletal relationships are therefore required.

Nevertheless, careful accumulation of anatomical descriptions, such as those presented in this study, is essential for establishing an objective understanding of the structural characteristics associated with malocclusion. Such anatomical evidence may gradually provide a more reliable foundation for interpreting clinical manifestations and for developing future orthodontic diagnosis and treatment strategies.

## 5 Limitations

Several limitations should be acknowledged. First, the study population consisted of patients undergoing orthognathic surgery combined with orthodontic treatment, and substantial inter-individual variation may exist within each skeletal class. Second, vertical skeletal patterns were not analyzed because of the limited sample size within each anteroposterior group. Third, the mid-sagittal plane was defined using nasion and crista galli; however, deviation of the crista galli is common and may have affected measurement accuracy.

In addition, CT image orientation was based on the occlusal plane, which may introduce variability, as standardization of scan orientation remains a limitation in this field. Furthermore, the boundary between the tongue and the palatal mucosa was indistinct in some cases, which may have affected measurement accuracy.

## 6 Conclusion

In this study, palatal mucosal thickness (PMT) and bony palatal dimensions were evaluated in relation to anteroposterior jaw relationships in the female orthognathic surgery candidates.

PMT exhibited substantial inter-individual variation, and clear differences attributable to palatal morphology were not identified. PMT showed no difference between age groups, and may serve as a representative indicator of palatal mucosal structure. A weak positive correlation was observed between mucosal thickness and palatal depth at P1; that is, a deeper palate at P1 was associated with thicker mucosa.

The differences in palate dimensions were evident between Class II and Class III, but the difference was not distinct between Class I and the other two Classes. Class III exhibited greater palatal width than Class II, while palatal length was shorter. Palatal depth at P1 was greater in Class II than in Class III, whereas no inter-class difference was found at M1.

## Data Availability

The datasets presented in this article are not readily available because the raw data supporting the conclusions of this article will be made available by the authors, without undue reservation. Requests to access the datasets should be directed to Yusuke Suzuki, suzuki.yuusuke62@nihon-u.ac.jp.
